# *Leiurus quinquestratus* venom promotes β islets regeneration and restores glucose level in streptozotocin induced type 2 diabetes mellitus in rats

**DOI:** 10.1038/s41598-025-94030-0

**Published:** 2025-04-07

**Authors:** Wesam M. Salama, Sabry A. El-Naggar, Ghada A. Tabl, Nabila I. El-Desouki, Lamiaa M. El Shefiey

**Affiliations:** Zoology Department, Faculty of Science, Tanta University, Tanta, Egypt

**Keywords:** Diabetes mellitus, Insulin, Metformin, Scorpion, *Leiurus quinquestriatus*, Venom, Antioxidant, Pancreatic tissues, Drug discovery, Physiology, Zoology, Diseases

## Abstract

Diabetes mellitus type 2 (T2-DM) is one of the most prevalent chronic metabolic diseases, marked by insulin resistance and a relative lack of insulin production. T2-DM can be treated using various methods; however, these treatments are risky for several vital organs. Subsequently, novel T2-DM replacement therapies should be discovered. The goal of this study was to see how efficient *Leiurus quinquestratus* venom (LQV) was as a diabetic medicine for the treatment of T2-DM in rats. The median lethal dose (LD50) of LQV has been determined. Then, forty male Sprague Dawley rats were divided into four groups (*n* = 10) as follows, with group 1 (Gp1) separated as a negative control. Gp2, Gp3, and Gp4 were fed a high-fat diet (HFD) for 12 weeks before receiving an intraperitoneal (i.p) injection of streptozotocin (STZ) as 30 mg/kg b.wt. Gp3 received metformin (Met) as 150 mg/kg b.wt i.p. LQV as 1/40 LD50 was given i.p. to Gp4. Treatments with Met or LQV were once every day for eight weeks. Hematological, biochemical, histopathological, and immunohistochemical studies were determined, along with the percentages of changes in total body weight. Results: LD50 of LQV was 0.3 mg/kg b.wt. Met or LQV treatment reduced hyperglycemia and C-peptide levels and lessened the hepato-renal biomarkers disorders in T2-DM rats. Intriguingly, histological analysis revealed that LQV treatment outperformed Met in improving and restoring β-cells in pancreatic tissues of T2-DM mice. In conclusion, this study demonstrated a new and promising method for treating T2-DM with LQV. Further investigation is required to isolate the bioactive elements from LQV to treat T2-DM.

## Introduction

A complicated endocrine and metabolic condition that can lead to problems and organ damage is diabetes mellitus (DM). These complications led to chronic inflammation, hyperinsulinemia, insulin resistance, or oxidative stress^1^. Among DM types, type 2 diabetes mellitus (T2-DM) is caused by a defect in insulin secretion by pancreatic β-cells and the inability of insulin^2^. Microvascular and macrovascular problems of T2-DM significantly increase the risk of cardiovascular disease, end-stage renal disease, retinopathy, and neuropathy^3^. To treat T2-DM, oral hypoglycemic drugs such as metformin (Met), thiazolidinediones, alpha-glutamyl transferases, and sulphonyl urease are frequently utilized. These treatments come with a risk of anemia, hypoglycemia, tiredness, and diarrhea^4^. Most T2-DM patients continue to get Met as their first line of treatment; Met is commonly chosen as a comparative standard reference drug in T2-DM studies. Also, it is used due to its established efficacy, safety, and widespread use. A lot of research showed the adverse side effects of antidiabetic drugs, for instance, hypoglycemia, weight loss, diarrhea, and gastrointestinal disturbance. As a result, second-line or other alternative treatment approaches must be tailored to the specifics of each patient^5^.

Researchers are increasingly moving toward the creation of new antidiabetic medications from natural products targeting pathways or components linked with DM since they are cheaper and have fewer side effects than traditional therapy^6^. In particular, natural substances obtained from animals have been used to control diabetes^7^. *Leiurus quinquestratus* scorpion belongs to the family Buthidae, the most harmful species for humans^8^. However, a recent study evaluated the antitumor effect of whole-body extract of *L. quinquestratus* scorpion^9^. Scorpion venom (SV) contains oligopeptides, nucleotides, amino acids, and other organic compounds and is water soluble.

Additionally, it includes low-molecular-weight chemicals like serotonin, histamine, and protease inhibitors, as well as enzymes like phospholipases and hyaluronidase. Therefore, SV may offer a promising approach for the development of new pharmaceuticals, such as anti-microbial, anti-leishmanial, anti-malarial, anti-arthritic, anti-inflammatory, anti-cancer, and cytotoxic effects^10–14^^.^ *Androctonus crassicauda* venom has been found to be a potential antidiabetic effect in T1-DM in animal models^15^. Additionally, a recent study reported that the whole-body *Scorpio maurus palmatus* extract considerably improved pancreatic islets^16^. Previous studies showed that the scorpion venom, in combination with gypsum, had antidiabetic activities in streptozotocin-induced diabetic mice^7^. A recent study ameliorated the histopathological and immunohistochemical changes in splenic tissues of T2-DM rats^17^. Also, the peptides from scorpion venom are supposed to maintain the β-cell in a depolarized state, which would prolong insulin secretion only in the presence of glucose^18^. Active polypeptide of LQV accelerates wound healing with a more potent anti-inflammation and antibacterial impact and may be a useful topical medication for the treatment of diabetic ulcers^19^. The current study sets out the effectiveness of LQV as an antidiabetic medication for the treatment of T2-DM in rats.

## Materials and methods

### Chemicals

Analytical grades were used for all chemicals and reagents. Met was obtained from Sigma-Aldrich (Berlin, Germany), and it was diluted with phosphate buffer saline (PBS) at a concentration of 150 mg/kg in 300 µl^20^. Sigma-Aldrich (USA) provided the streptozotocin (STZ) and the isoflurane (for anathesia). From the Bio Diagnostic Company in Egypt, kits for glucose, C-peptide, aspartate aminotransferase (AST), alanine aminotransferase (ALT), total protein, urea, creatinine, catalase, superoxide dismutase, and malondialdehyde were obtained.

### Preparation of *L. quinquestriatus* venom

Professional scorpion hunters gathered 100* L. quinquestriatus* scorpions from Aswan, Egypt. Scorpions were moved to the Invertebrate lab in the Zoology Department. Scorpions were milked by stimulating their telsons electrically (12–17 V), and the venom was subsequently lyophilized in Corporate Serum and Vaccine (VACSERA)^12^.

### SDS–PAGE profile of *L. quinquestriatus* venom

The sample was analyzed using 12% gel sodium dodecyl sulfate–polyacrylamide gel electrophoresis (SDS-PAGE) in accordance with Laemmeli^21^. With Coomassie Blue R-250 Silver 0.1%, proteins were stained. The molecular mass standard (Sigma, S8445) was calculated in parallel to get the molecular weights of the proteins. After the gel was photographed, its molecular weights were ascertained using Molecular Imaging Software (MIS, Kodak).

### Determination of the median lethal dose of *L. quinquestriatus* venom

Six groups (*n* = 6) of a total of 36 male rats were created. LQV was administered intraperitoneal (i.p) at various doses (0.1–5 mg/kg) to several groups. To determine the median lethal dosage (LD_50_), groups were observed for 24 h. The probit analysis was used to determine the LD_50_ value^22^.

### Normal balanced and high fat diets

The normal balanced diet (NBD), consisting of 10% protein, 10% fat, 74.4% carbs, 3.5% mineral mixture, 0.1% methionine, 1% vitamin mixture, and 1% fiber, was used to feed healthy control rats. The high-fat diet (HFD), consisting of 64 g of normal chow, 32 g of animal-sourced saturated fat, 300 IU of vitamin D3, and 15% and 12% of cholesterol, was used to treat rats.

### Experimental animals

Male rats were transferred to the laboratory; their weights were between (120 ± 5) g. The rats were kept in cages at room temperature (25 °C) with a constant 12-h light/dark cycle, and they had free access to their regular meal and water. In managing the laboratory animals, the National Institutes of Health’s standards for the care and use of laboratory animals and the recommendations of the National Research Centre Ethics Committee were followed. The work was authorized by the Institutional Animal Care Committee (IACUC-SCI-TU 0181). All the experiments were performed in accordance with relevant ARRIVE guidelines 2.0 in handling and euthanasia of animals. The choice of male rats in diabetic studies is justified by the observed sexual dimorphism in insulin sensitivity, susceptibility to diabetes, and the physiological response to high-fat and high-sucrose diets. Research has shown that male rats exhibit higher hyperglycemia and lower serum insulin concentration compared to females, making them a relevant model for studying diabetes^23^.

### Induction of T2-DM in rats

After 12 weeks of HFD feeding, group 1 (Gp1) and Gp2 were given a single injection of STZ at a dose of 30 mg/kg i.p. after an overnight fast 2. The use of 30mg/kg STZ in this study may help induce diabetes in rats without causing severe toxicity or mortality, allowing for the investigation of the pathophysiology and treatment of diabetes. Also, it produces moderate hyperglycemia and is considered sub-diabetogenic, making it suitable for inducing type 2 diabetes in rats^24^. Blood was collected from the tail vein three days after the STZ injection; the glucose level was determined using a portable glucometer (One Touch Select, Life scan, Inc., California, USA). T2-DM rats were defined as animals with an FBS concentration greater than 250.

### Experimental protocol

Forty male Sprague Dawley rats were split into four groups (*n* = 10) as follows: group 1 (Gp1) remained as a negative control. Gp2, Gp3, and Gp4 were given a high-fat diet (HFD) for 12 weeks before receiving an intraperitoneal (i.p) injection of streptozotocin (STZ) as 30 mg/kg b.wt. Gp3 received metformin (Met) as 150 mg/kg b.wt i.p. LQV as 1/40 LD_50_ was given i.p. to Gp4^25^.Treatments with Met or LQV were once every day for eight weeks. Rats were given 2% isoflurane anesthesia. For hematological parameters, blood samples were taken. For biochemical examination, sera samples and 10% liver homogenate were kept at -20 °C. For histological analysis, pancreas tissues were fixed in buffered formalin.

### Determination of the percentage of total body weight changes

Every group of rats was given a weight, and the equation that follows was used to determine the percentage change in the total body weight (% b.wt): (final b.wt − initial b.wt/initial b.wt) × 100.

### Determination of hematological and biochemical parameters

The electronic blood counter (Menday, China) was used to measure the following parameters from fresh blood samples: red blood cells (R.B.Cs), hemoglobin content (Hb), hematocrit (Hct%), platelets, white blood cells (W.B.Cs), and differential counts lymphocyte, monocyte, and neutrophil. The serum total protein, glucose, and C-peptide were measured at^25–28^^.^ ALT and AST activities were assessed^29^. Creatinine and urea levels were measured according to Newman and Price^30^. SOD activity was measured in accordance with Nishikimi et al.^31^; CAT activity was measured following the approach of Aebi^32^. Based on the techniques previously published by Li et al.^33^, MDA was evaluated.

### Histopathological investigation

Pancreatic tissue samples were collected and fixed in 10% formalin, according to Bancroft and Gamble^34^. Using microtomes, sections of pancreatic tissues (5 mm) were cut from paraffin blocks, stained with hematoxylin and eosin, and viewed using an Optica light microscope (B-350). An immunohistochemical reaction was performed using the avidin–biotin method as described by Hsu et al.^35^. The anti-insulin antibody intensity in pancreatic islets was measured using Image J software.

### Statistical analysis

Data entry and analysis were performed on the computer using the IBM SPSS® software program, version 16.0, USA. S.E. was employed in the data analysis, along with numerical values and percentages. The F-test (ANOVA) and Post Hoc test were used to compare the groups (LSD) when the data were evenly distributed, with significance being reached at *p* < 0.05.

## Results

### *Protein profiling and LD*_*50*_* of L. quinquestratus venom*

**Fig. 1:**
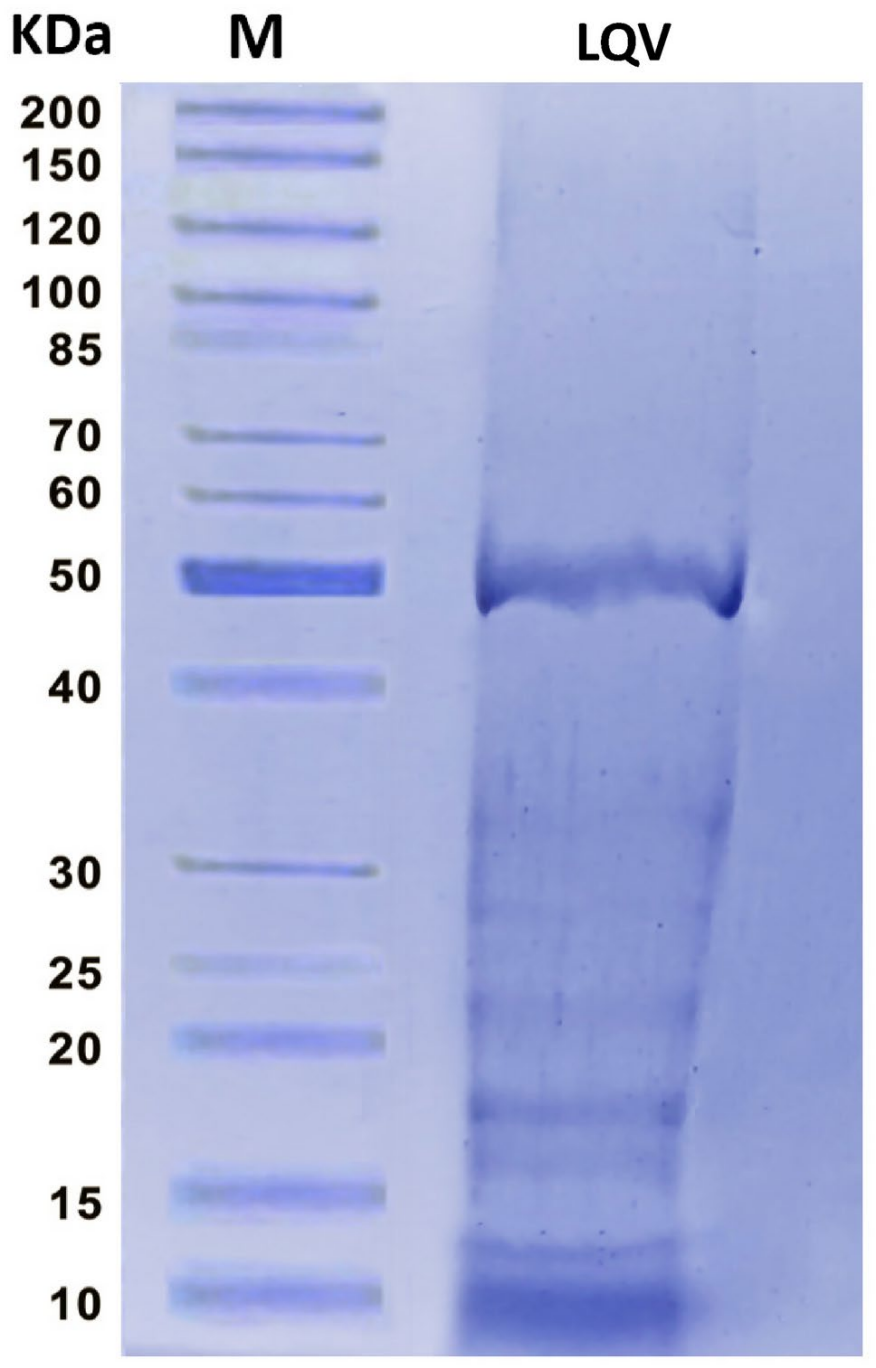
Sodium dodecyl sulfate–polyacrylamide gel electrophoresis analysis of LQV. M: protein standard marker (10—200 kDa, Biolaps, England).

**Fig. 2:**
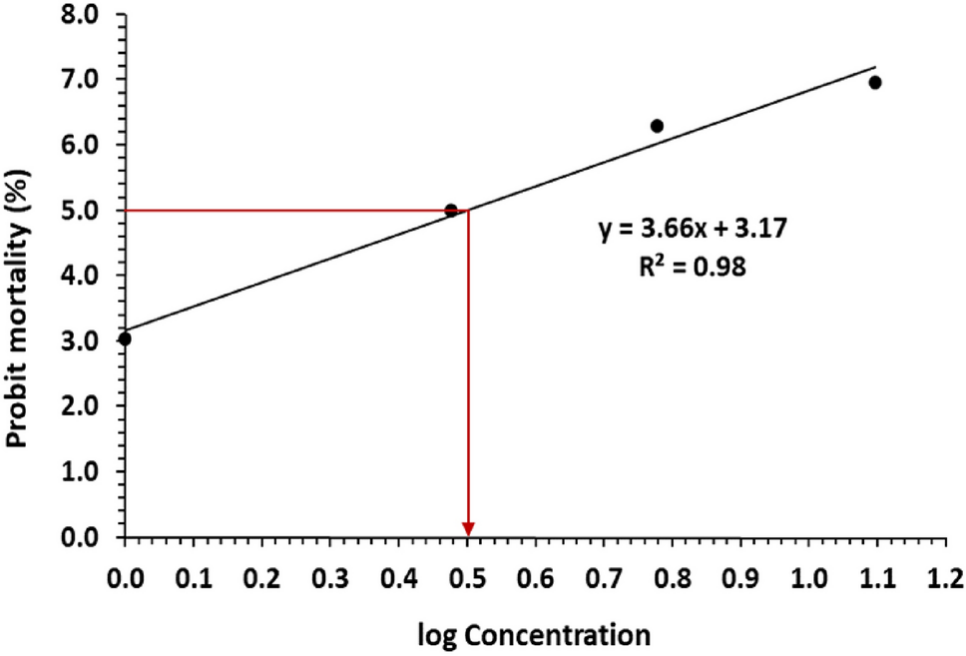
The median lethal dose (LD_50_) of LQV on rats after 24 h using probit analysis.

The SDS-PAGE profile analysis of LQV showed the presence of eight protein bands with molecular weights of 10, 12, 16, 18, 22, 27, 33 and 50 kDa (Fig. 1). The LD50 value of LQV that killed 50% of rats was 0.3 mg/kg b.wt (Fig. 2).

### Treatment with *L. quinquestratus* venom restored the percentages of body weight changes

**Fig. 3:**
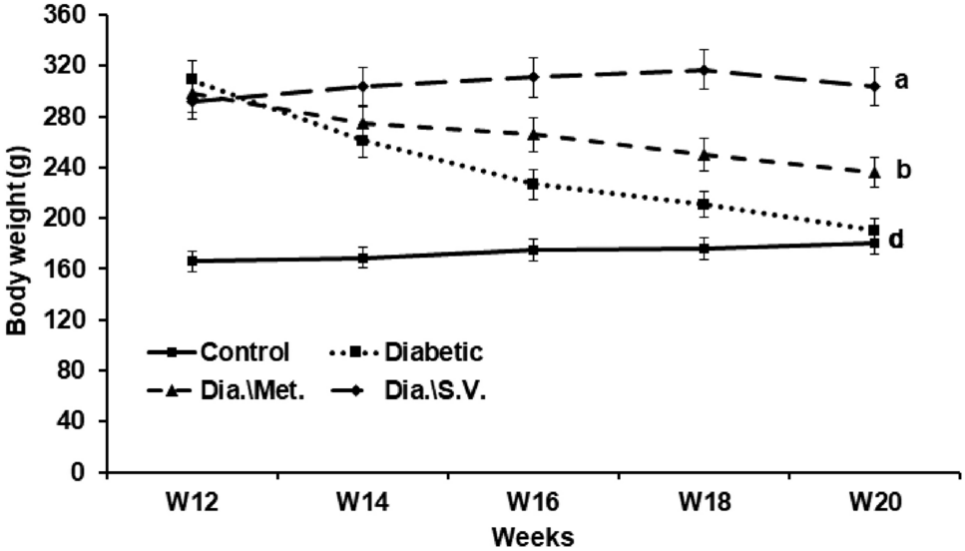
Kinetic in body weight changes of different groups. The means that do not share the same letter are significantly different (*p* < 0.05).

The results showed that the percentages of body weight (% b.wt) change was significantly decreased in Gp2 (T2-DM rats) (-38.56%) when compared to Gp1 (Control) (*p* < 0.05). The % b.wt change of T2-DM rats that were treated with Met (Gp3) was -20.74%, while this percentage was 4.07% in the T2-DM rats that were treated with LQV (Gp4) (Fig. 3).

### Treatment with *L. quinquestratus* venom ameliorated the hematological changes in T2-DM rats

The R.B.Cs count, Hb concentration, and Hct % were significantly decreased in the T2-DM group (Gp2) when compared to their control (*p* < 0.05). As compared to Gp1, the platelet count significantly decreased in Gp2. The treatment of T2-DM groups with Met (Gp3) or LQV (Gp4) led to improvement in the alterations that were induced in the hematological parameters of Gp2 (Table 1). The W.B.Cs count, and their differential percentages significantly increased in Gp2 compared to their control groups. Treatment with Met/ LQV of Gp3 or Gp4 significantly decreased the total W.B.Cs count and their differential percentages close to Gp1 (Table 2).

**Table 1:** The total count of R.B.Cs, Hb concentration, %Hct, and the total platelets count of different groups.

Groups	R.B.Cs (× 10^6^/µL)	Hb (g/dL)	Hct (%)	Platelets (× 10^3^/µL)
Ctrl	5.55 ± 0.67^**a**^	13.53 ± 0.30^**b**^	30.58 ± 0.67^**a**^	636.5 ± 20.95^**a**^
Diabetic	4.31 ± 0.29^**b**^	11.51 ± 0.22^**c**^	24.98 ± 2.72^**b**^	403.75 ± 4.27^**c**^
D.\Met	5.10 ± 0.36^**a,b**^	13.05 ± 0.19^**b**^	30.78 ± 1.72^**a**^	556.75 ± 9.54^**b**^
D.\LQV	5.44 ± 0.18^**a**^	14.35 ± 0.24^**a**^	31.55 ± 0.70^**a**^	614.0 ± 35.36^**a**^
F-Value	57.23	98.29	12.95	97.83
P-Value	0.005	< 0.001	< 0.001	< 0.001

The values represented mean ± SD. Ctrl: Control group; Met: Metformin; LQV: *Leiurus quinquestratus* venom; D: Diabetic; R.B.Cs: Red blood cells; Hb: Hemoglobin; Hct: Hematocrit. *P*-value < 0.05 was statistically significant. The means that do not share the same letter are significantly different (Tukey’s test).

**Table 2:** The total count of white blood cells and the differential percentages of different groups.

Groups	W.B.Cs (× 10^3^/µL)	Monocytes (%)	Lymphocytes (%)	Neutrophils (%)
Ctrl	6.75 ± 0.13^**b**^	5.75 ± 0.50^**c**^	70.75 ± 2.06^**a**^	20.25 ± 1.71^**b**^
Diabetic	9.60 ± 0.22^**a**^	8.95 ± 0.50^**a**^	37.00 ± 0.82^**b**^	52.00 ± 0.82^**a**^
D.\Met	7.13 ± 0.31^**b**^	6.75 ± 0.96^**b**^	74.00 ± 4.55^**a**^	17.50 ± 1.29^**b,c**^
D.\LQV	6.93 ± 0.46^**b**^	6.00 ± 0.01^**b,c**^	75.50 ± 1.29^**a**^	16.75 ± 2.22^**c**^
F-Value	77.10	30.78	196.7	452.9
P-Value	< 0.001	< 0.001	< 0.001	< 0.001

The values represented mean ± SD. Ctrl: Control group; Met: Metformin; LQV: *Leiurus quinquestratus* venom; D: Diabetic; W.B.Cs: White blood cells. *P*-value < 0.05 was statistically significant.

### Treatment with Met or *L. quinquestratus* venom decreased glucose and C-peptide levels in T2-DM rats.

**Fig. 4:**
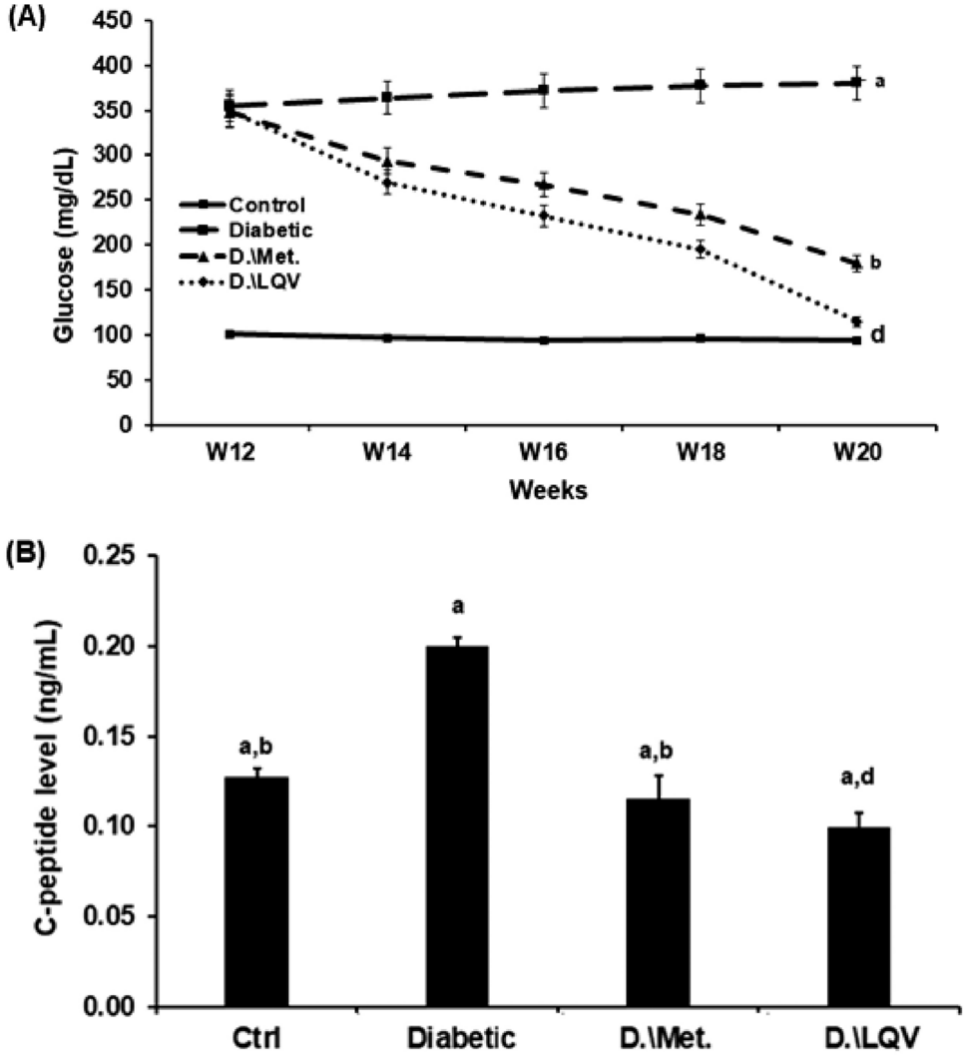
Kinetic of glucose (**A**) and C-peptide levels (**B**) in the different groups. Means that do not share the same letter are significantly different (*p* < 0.05).

The results showed that there was a significant increase in the glucose level in the T2-DM rats (Gp2) when compared to Gp1. Treatment with Met or LQV significantly decreased the blood glucose levels when compared to Gp2 (Fig. 4A). In Gp2, the level of C-peptide was significantly increased when compared to their control (*p* < 0.05). The administration of Met or LQV in Gp3 and Gp4 decreased the level of C-peptide when compared to T2-DM rats alone (Fig. 4B).

### Treatment with *L. quinquestratus* venom ameliorated the biochemical alterations induced in T2-DM rats

**Fig. 5:**
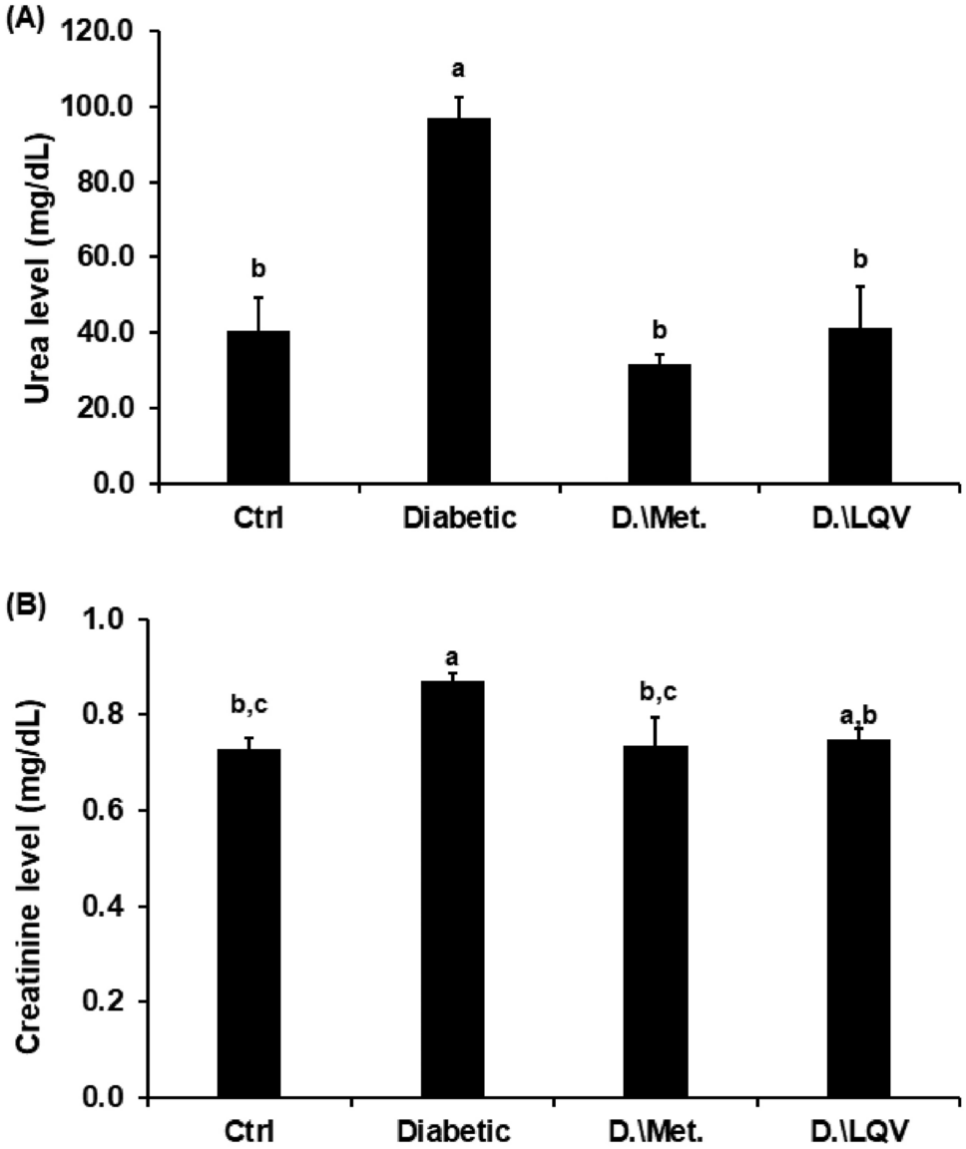
Serum urea and creatinine levels in the different groups. The values represented mean ± SD. Ctrl: Control group; Met: Metformin; LQV: *Leiurus quinquestratus* venom; D: Diabetic; *P*-value < 0.05 was considered to be statistically significant. The means that do not share the same letter are significantly different (Tukey’s test).

Significant increase in the liver enzymes (ALT and AST) activities, kidney biomarkers (urea and creatinine) levels, and a significant decrease of total protein in T2-DM rats (Gp2) when compared to their values in Gp1 (*p* < 0.05). Treatment of Gp3 or Gp4 with Met or LQV led to improvement in hepatorenal function evidenced by a significant decrease of ALT, AST activities, urea, and creatinine levels, and a significant increase in serum total protein (Table 3 and Fig. 5). Significant decreases in the activities of the antioxidant enzymes in hepatic tissue (SOD and CAT) were reported in Gp2 when compared to the control group (*p* < 0.05). In contrast, the level of MDA in hepatic tissue was significantly increased due to lipid peroxidation that was induced by oxidative stress in the T2-DM rats. Treatment of Gp3 and Gp4 with Met or LQV improved the antioxidant/oxidant biomarkers when compared to the T2-DM rats only (Table 4).

**Table 3:** Serum ALT, AST activities, and total protein level in different groups.

Groups	ALT (U/L)	AST (U/L)	Total protein (g/dL)
Ctrl	42.0 ± 2.16 ^**b**^	78.50 ± 5.55 ^**b**^	8.78 ± 0.19 ^**a**^
Diabetic	58.8 ± 2.99 ^**a**^	130.25 ± 11.90 ^**a**^	6.25 ± 0.05 ^b^
D.\Met	46.5 ± 5.07 ^**b**^	93.23 ± 4.30 ^**b**^	8.38 ± 0.28 ^**a**^
D.\LQV	33.0 ± 1.83 ^**c**^	80.5 ± 7.87 ^**b**^	8.42 ± 0.21 ^**a**^
F-Value	43.12	36.42	131.9
P-Value	< 0.001	< 0.001	< 0.001

The values represented mean ± SD. Ctrl: Control group; Met: Metformin; LQV: *Leiurus quinquestratus* venom; D: Diabetic; ALT: Alanine transaminase; AST: Aspartate transaminase. *P*-value < 0.05 was statistically significant. The means that do not share the same letter are significantly different (Tukey’s test).

**Table 4:** Malondialdehyde levels, superoxide dismutase, and catalase activities in the different groups.

Groups	SOD (U/mg tissue)	CAT (U/mg tissue)	MDA (nmol/mg tissue)
Ctrl	9.89 ± 0.74^**a**^	82.86 ± 1.16^**a**^	34.29 ± 1.34^**d**^
Diabetic	5.85 ± 1.07^**c**^	51.04 ± 2.13^**d**^	81.91 ± 2.16^**a**^
D./Met	7.51 ± 0.82^**b,c**^	70.85 ± 2.70^**c**^	49.92 ± 1.98^**b**^
D.\LQV	8.88 ± 0.45^**a,b**^	77.20 ± 1.95^**b**^	44.24 ± 0.73^**c**^
F-Value	19.12	181.1	621.3
P-Value	< 0.001	< 0.001	< 0.001

The values represented mean ± SD. Ctrl: Control group; Met: Metformin; LQV: *Leiurus quinquestratus* venom; D: Diabetic; SOD: Superoxide dismutase; CAT: Catalase; MDA: malondialdehyde. *P*-value < 0.05 was statistically significant. The means that do not share the same letter are significantly different (Tukey’s test).

### Treatment with *L. quinquestratus* venom ameliorates histopathological changes in pancreatic tissues of T2-DM rats

**Fig. 6:**
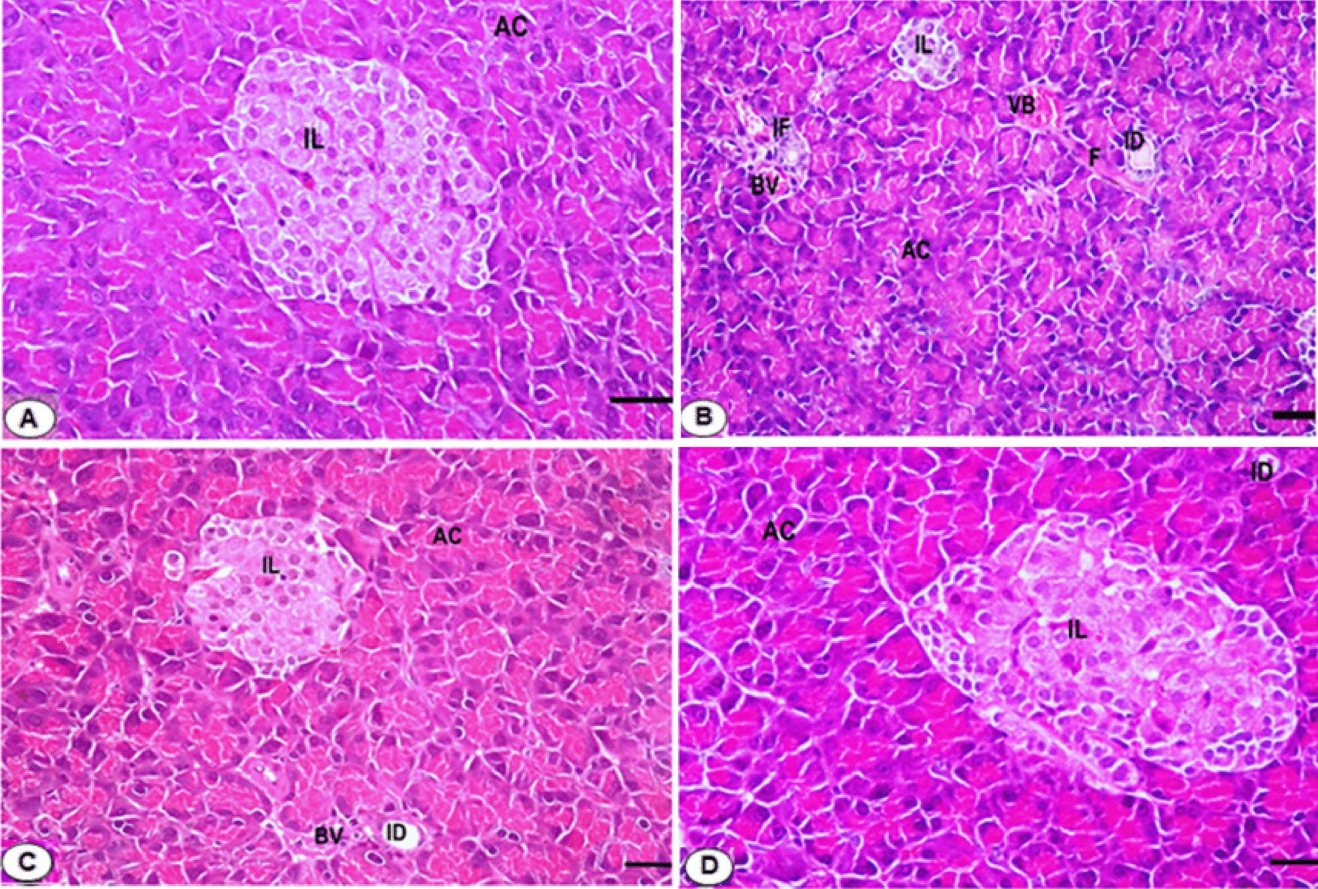
(**A**–**D**) Photomicrographs of pancreatic sections stained with H&E (**A**). Section of Gp1 )ctrl (rats showing normal architecture of acini (AC) and islets of Langerhans (IL). (**B**) Section of Gp2 (Diabetic) rats showing cytoplasmic vacuolation (arrows) in most of the islet cells (IL), necrotic areas (NA) represented as arrow, disorganized acinar cells (AC), congested and dilated blood vessel (BV), Infiltration of the inflammatory leucocytes (IF). (**C**) Section of Gp3 (D/Met) rats treated with Met (150 mg/Kg) showing partial improvement of the cells in the islet of Langerhans (IL), a recovery of few acinar cells (AC) to normal form, clearly reduction of the accumulated fibers, decline of the dilated blood vessels congested (BV). (**D**) Section of Gp4 (D/LQV) rats treated with LQV (0.0075 mg/kg) showing an obvious recovery of the arrangement of the normal pancreatic islet cells (IL), acinar cells (AC), completely disappearance of the accumulated fibers and no seen congested blood vessels (BV). H&E, scale bar = 6.25 μm.

**Fig. 7:**
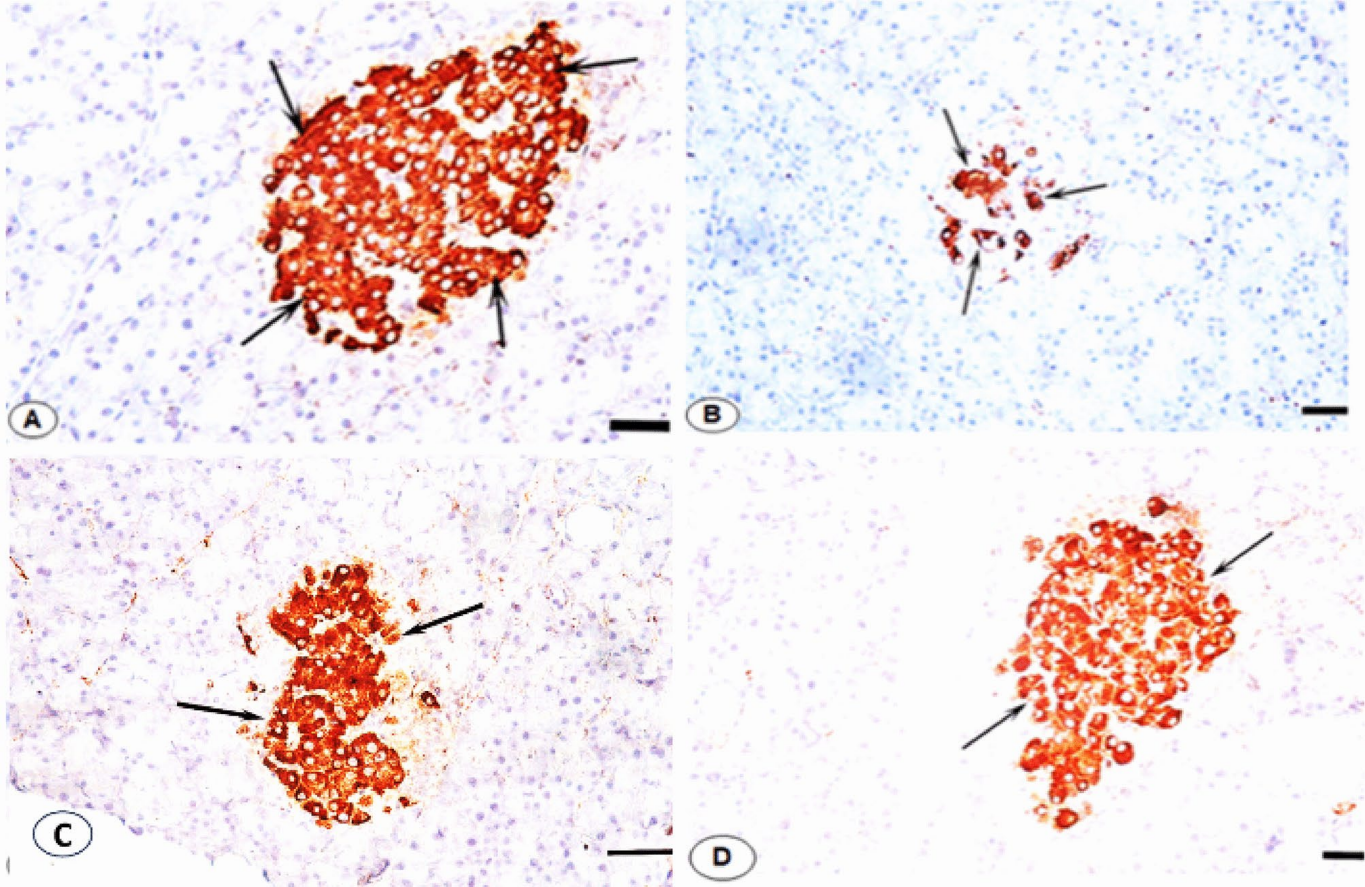
(**A–D**) Photomicrographs of pancreatic sections showing anti-insulin immunostaining. (**A**) Sections of Gp1 (Ctrl) rats showing a strong expression of insulin secreting β-cells (arrows) as a dark brown granulated β-cells cytoplasm. (**B**) Sections of Gp2 (Diabetic) rats demonstrating an obvious reduction of the expression of insulin secreting β -cells (arrows). (**C**) Sections of Gp3 (D/Met) rats treated with Met (150 mg/kg) daily for 8 weeks showing an improvement in the expression of immunostain to insulin secreting β-cells (arrows). (**D**) Sections of Gp4 (D/LQV) rats treated with LQV (0.0075 mg/kg) daily for 8 weeks showing a marked recovery of the strong expression of immunostain to insulin secreting β-cells (arrows). scale bar = 6.25 μm.

**Fig. 8:**
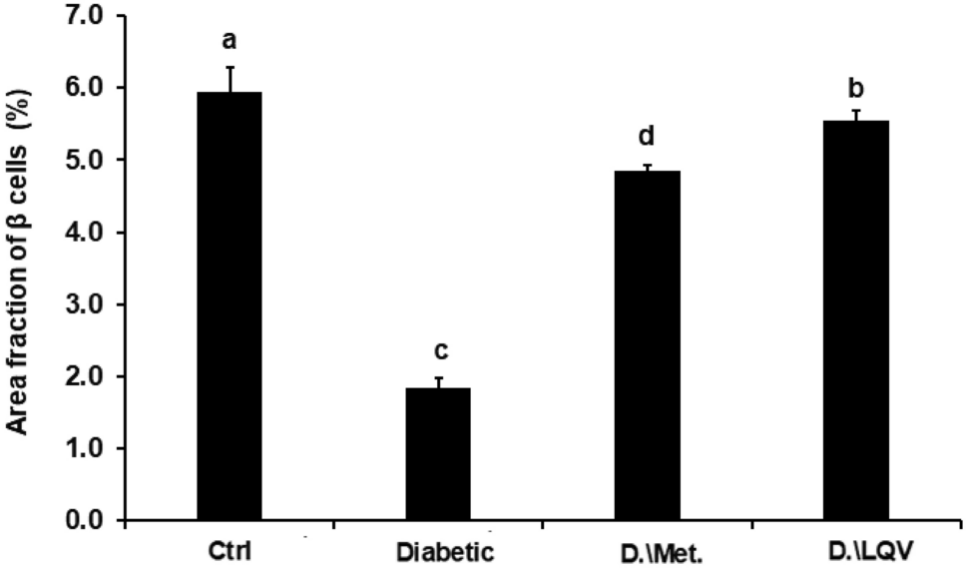
Morphometric measurements showing changes in the mean values of area of insulin reactive beta-cells (%).

The islet of Langerhans and pancreatic acini displayed normal architecture in the pancreatic tissue of Gp1 stained with H&E. The pyramidal cells that make up the pancreatic acini have rounded basal nuclei. Each pyramidal cell has a basophilic basal region and acidophilic granules in the apical region. The Islets of Langerhans are rounded or oval in shape, dispersed among the acini, and appear slightly stained with H and E (Fig. 6A). Necrotic areas, islet of Langerhans cell degeneration, acinar cell disarray, multiple blood vessel congestions, dilated intercalated pancreatic ducts, and accumulated fibers peripheral to blood vessels were all visible in the pancreatic tissues of T2-DM rats (Gp2) (Fig. 6B). The pancreatic tissues of T2-DM rats administered Met (Gp3) exhibited some amelioration in the islet of Langerhans cells, a return of some acinar cells to normal form, a decrease in the accumulated fibers, and detection of dilated congested blood vessels with the majority of intercalated ducts visible (Fig. 6C). When T2-DM rats were given LQV (Gp4), there was a clear return to the normal arrangement of the pancreatic islet and acinar cells. No dilated blood vessels, intercalated ducts, or fiber accumulations are visible (Fig. 6D). The β-cells that secrete insulin exhibited a strong and normal immunoreactivity in the pancreatic sections of the control rats (Gp1). Dark brown granules were observed in the cytoplasm of β-cells, indicating positive insulin expression (Figs. 7A and 8). The area percentage of β-cells was significantly lower in T2-DM rats than in normal control rats (*p* < 0.001). Figures 7B and 8 of the T2-DM (Gp2) rats’ pancreatic sections showed a clear decrease in the immunostaining of the insulin-secreting β-cells in the islets of Langerhans. The expression of insulin-secreting β-cells in the islets of Langerhans was partially improved in pancreatic sections of T2-DM rats treated with Met (Figs. 7C and 8). Pancreatic sections from LQV-treated T2-DM rats clearly demonstrate recovery (Figs. 7D and 8).

## Discussion

According to this study, the protein profile of LQV had eight bands with molecular weights varying from 10 to 50 kDa. This result agreed with studies by Salama and Sharshar and Akbar et al.^36,37^. Previous research that examined SV using SDS-PAGE identified twelve distinct bands with molecular weights of 140, 70, 50, 33, 30, 27, 22, 18, 14, and 10 kDa, as well as two bands smaller than 5 kDa^38^. This study showed that LQV had an LD50 of 0.3 mg/kg b.wt. According to Salama^39^, the LD50 was 0.25 mg/g b.wt. T2-DM rats showed a significant reduction in the % b.wt change. However, the treatment of the diabetic rats with Met or LQV decreased the changes in the b.wt. This improvement may be attributable to the alleviation of metabolic abnormalities brought on by diabetes.

The T2-DM group had considerably lower R.B.Cs count, Hb level, Hct value, and platelet count. In addition, the T2-DM group had a significantly high W.B.C. count. A previous study showed that the Hb, Hct, and R.B.Cs count levels were low when compared to those of the control group.

In order to determine how Met or LQV administration affected the levels of hematological parameters in T2-DM-induced rats, this study assessed that effect. These modifications of the hematological parameters improved when T2-DM groups were treated with Met or LQV.

This suggests that Met or LQV can stimulate erythropoietin formation, which in turn stimulates bone marrow stem cells to produce blood cells. A previous study showed that the treatment with *Tityus serrulatus* venom increased the total R.B.Cs count, Hb level, and Hct value in rats^40^.

Both glucose and C-peptide levels were significantly elevated in T2-DM rats. In T2-DM, pancreatic β-cells had a detrimental effect that increased glucose and C-peptide levels. Following T2-DM induction, the administration of Met or LQV restored the levels of glucose and C-peptide. Earlier studies showed that Met lowers blood glucose levels by inhibiting intestinal glucose absorption, suppressing hepatic glucose synthesis, decreasing hepatic glucose output, facilitating cellular glucose uptake, and increasing insulin sensitivity^41^.

Moreover, Met promotes GLUT-4 transporter translocation to the plasma membrane and stimulates glucagon-like peptide-1 (GLP-1) to increase glucose absorption in skeletal muscle^42^. Met also lowers blood sugar by enhancing the activation of the insulin receptor and its substrate, which boosts the uptake of glucose in the liver cell^43^. It controls abnormally high blood glucose levels and reduces associated diabetic complications by reducing hepatic gluconeogenesis and increasing insulin sensitivity. It also exhibits strong anti-inflammatory, anti-oxidative, and anti-apoptotic properties^44^. A previous study on scorpion venom in combination with gypsum in diabetic mice up-regulated peroxisome proliferator-activated receptor gamma and pancreatic and duodenal homeobox one expression in the pancreas^7^. Also, a similar finding in regard to the anti-diabetic effect of Iranian snake venom was reported^42^.

In T2-DM rats, the level of total protein was decreased, and the activity of ALT, AST, urea, and creatinine levels was increased. In T2-DM rats, the activities of ALT and AST were significantly raised, while total protein was reduced, according to Kim et al.^45^. T2-DM negatively impacts many organs, including the kidney, causing systemic problems^46^. Improvements in hepato-renal functioning were observed in T2-DM rats treated with Met or LQV, as shown by a significant decrease in liver enzymes and kidney biomarkers. Earlier studies showed that Met therapy ameliorates the hepato-renal dysfunctions brought on by T2-DM rats^47,48^. Also, a previous study reported that upon the injection of LQV, the activity of the liver enzymes ALT and AST dramatically improved^40^.

Diabetes mellitus, with an emphasis on T2-DM, processes involved in hyperglycemia-induced oxidative stress^6^. Accordingly, SOD and CAT activities significantly decreased, while the MDA level increased considerably. A previous report showed that in T2-DM rats, the SOD and CAT activities significantly decreased, while MDA levels had significantly increased^49^.

The antioxidant/oxidant hemostasis in T2-DM rats treated with Met or LQV improved. After treatment of T2-DM rats with Met or LQV, SOD, and CAT activities were significantly increased while the MDA level was decreased compared to their values in T2-DM rats. These results were consistent with an earlier study showing that Met reduces oxidative stress brought on T2-DM rats^50^. Bradykinin potentiating factor (BPF) was isolated from LQV and showed a potent antioxidant potential that can lessen the liver’s toxicity induced by carbon tetrachloride in mice^51^.

The pancreatic tissue from control rats has a normal pancreatic tissue architecture. In contrast, necrotic regions, islets of Langerhans cell degeneration, acinar cell disarray, the formation of many congestions, and dilated blood vessels were visible in the pancreatic tissues of the T2-DM rats. Similar findings have been reported in a previous study, which showed that T2-DM rats’ pancreas had fewer islets and/or less β-cell volume/mass, as well as morphological abnormalities to many β-cell organelles^52^. Furthermore, previous investigations documented several histological changes in the pancreatic tissues of T2-DM rats^53^. The islet of Langerhans cells in T2-DM rats that had been treated with Met showed some improvement, a few acinar cells returned to their normal state, and accumulated fibers were reduced. The protective effect of LQV could be due to its high antioxidant characteristics, which in turn benefits pancreatic β-cell functioning^16^.

The findings demonstrated that typical robust immunoreactivity to the insulin-secreting cells was expressed in the pancreas of normal control rats during IHC observations. Dark brown granules seen in the cytoplasm of β-cells are a sign of positive insulin expression. The immunostaining to the insulin-secreting β-cells in the islets of Langerhans was noticeably reduced in the T2-DM rats. The malfunction and death of pancreatic β-cells are important causes of DM. The deterioration of glucose homeostasis is caused by a decrease in insulin production in T2-DM, which is brought on by either decreased β-cell mass or altered β-cell function^54^. A previous study of obese, hyperglycemic rats showed a decline in the immune-stained pancreatic cell number, indicating the islets were losing their capacity to release insulin effectively^55^. The islets of Langerhans in the T2-DM rats treated with Met improved, and the islets of Langerhans in the LQV rats clearly recovered. This was demonstrated by an increase in the expression of immunoreactivity to the insulin-secreting islets of Langerhans in the diabetic islets of Langerhans.

Scorpion venom active polypeptide has a better anti-inflammatory and antibacterial effect, shortens the healing process of wounds, and maybe a novel and potent topical medication for the treatment of diabetic ulcers^56^. This is consistent with earlier studies that demonstrated that Brazilian S.V. promotes the proliferation of pancreatic β -cells, which could be useful in the treatment of T2-DM^57,58^.

## Conclusion

Based on the potential results that have been obtained from this study, it was found that the treatment of T2-DM rats with LQV potentially decreased glucose levels and enhanced pancreatic functionality. Further studies could be carried out to estimate novel peptides isolated and identified from LQV as promising anti-diabetic agents.

## Institutional review board statement

The study was conducted according to the guidelines of ARRIVE and approved by Ethical committee at the Faculty of Science, Tanta University.

## Supplementary Information

Supplementary Information 1.

Supplementary Information 2.

## Data Availability

The data that support the findings of this study are available from the corresponding author Salama, W, but restrictions apply to the availability of these data, which were used under license for the current study, and so are not publicly available. Data are available from the corresponding author upon reasonable request. No human participants were used in the current study.
