# Supplementary figures and images for: Leiurus quinquestratus venom promotes β islets regeneration and restores glucose level in streptozotocin induced type 2 diabetes mellitus in rats
Source: Sci Rep. 2025 Apr 7;15:11841. doi: 10.1038/s41598-025-94030-0 (PMC11976942; doi:10.1038/s41598-025-94030-0)

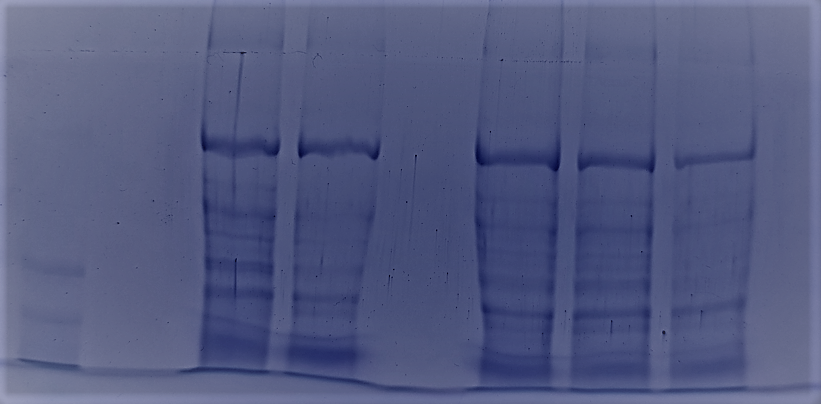

Supplement: Supplementary file 2 — Supplementary Information 2. [file 41598_2025_94030_MOESM2_ESM.png]
